# Management of Myeloma Bone Lesions

**DOI:** 10.3390/ijms22073389

**Published:** 2021-03-25

**Authors:** Jeng-Shiun Du, Chia-Hung Yen, Chin-Mu Hsu, Hui-Hua Hsiao

**Affiliations:** 1 Division of Hematology and Oncology, Department of Internal Medicine, Kaohsiung Medical University Hospital, Kaohsiung 80756, Taiwan; ashiun@gmail.com (J.-S.D.); e12013@gmail.com (C.-M.H.); 2 Graduate Institute of Clinical Medicine, College of Medicine, Kaohsiung Medical University, Kaohsiung 80708, Taiwan; 3 Graduate Institute of Natural Products, College of Pharmacy, Kaohsiung Medical University, Kaohsiung 80708, Taiwan; 4 National Natural Product Libraries and High-Throughput Screening Core Facility, Kaohsiung Medical University, Kaohsiung 80708, Taiwan; chyen@kmu.edu.tw; 5 Department of Medical Research, Kaohsiung Medical University Hospital, Kaohsiung 80708, Taiwan; 6 Center for Cancer Research, Kaohsiung Medical University, Kaohsiung 80708, Taiwan; 7 Center for Liquid Biopsy and Cohort Research, Kaohsiung Medical University, Kaohsiung 80708, Taiwan; 8 Cancer Center, Kaohsiung Medical University Hospital, Kaohsiung 80756, Taiwan; 9 Faculty of Medicine, Kaohsiung Medical University, Kaohsiung 80708, Taiwan

**Keywords:** multiple myeloma, osteolytic bone disease, denosumab, bisphosphonates, Wnt inhibitors

## Abstract

Multiple myeloma (MM) is a B-cell neoplasm characterized by clonal plasma–cell proliferation. The survival and prognosis of this condition have been significantly improved by treatment with active anti-MM drugs such as bortezomib or lenalidomide. Further, the discovery of novel agents has recently paved the way for new areas of investigation. However, MM, including myeloma-related bone diseases, remains fatal. Bone disease or bone destruction in MM is a consequence of skeletal involvement with bone pain, spinal cord compression, and bone fracture resulting from osteolytic lesions. These consequences affect disease outcomes, including patients’ quality of life and survival. Several studies have sought to better understand MM bone disease (MBD) through the classification of its molecular mechanisms, including osteoclast activation and osteoblast inhibition. Bisphosphonates and the receptor activator of the nuclear factor-kappa B (NF-κB) ligand (RANKL) inhibitor, denosumab, prevent skeletal-related events in MM. In addition, several other bone-targeting agents, including bone-anabolic drugs, are currently used in preclinical and early clinical evaluations. This review summarizes the current knowledge of the pathogenesis of MBD and discusses novel agents that appear very promising and will soon enter clinical development.

## 1. Introduction

Multiple myeloma (MM) is the second most common blood malignancy. MM is typically characterized by the proliferation of plasma cells in the bone marrow, which produces a monoclonal immunoglobulin [1]. Most patients with MM exhibit signs or symptoms related to the accumulation of plasma cells in bone or organs, or kidney damage caused by immunoglobulin deposition [2]. The clinical presentation of MM includes calcium level elevation, renal insufficiency, anemia, and bone disease (abbreviated as CRAB). MM generally occurs in elderly individuals with a median age of 69 years at diagnosis. The median overall survival of patients with MM is 6 to 7 years. Survival is hindered by comorbidities and MM bone disease (MBD). Bone destruction with osteolytic lesions, osteopenia, or pathologic fractures is a sign of myeloma in up to 70 to 80% of cases. Over 80% of patients demonstrate skeletal-related events (SREs), such as vertebral compression fractures, which may result in cord compression, hypercalcemia, and pathologic fractures [3,4].

MBD is a serious complication of MM that affects the performance and survival of myeloma patients. Osteocytes and the related microenvironment appear to be crucial in the development of MBD. In addition to other factors, they contribute to pathogenesis, including increased osteoclast (OC) activity, enhanced osteoblast (OB) inhibition, bidirectional signaling to activate OCs and suppress OBs, and immunomodulation of the bone marrow microenvironment, and all this results in the deregulation of bone turnover as well as osteopenia and SREs. 

Newly identified pathways have created new opportunities to identify effective therapeutic agents and develop novel therapeutic strategies to prevent SREs. In this review, we discuss the mechanisms of MM-related SREs, and summarize current antiresorptive therapies such as bisphosphonate (BP) and the denosumab monoclonal antibody (moAb) -targeting receptor activator of nuclear factor-kappa B (NF-κB) ligand (RANKL) and anti-MM therapies, which include autologous stem cell transplantation (ASCT) and bortezomib-based regimens to prevent MBD. The review also discusses potential bone-anabolic agents that are currently in development using preclinical models. 

## 2. Pathophysiology

In normal physiological settings, the activities of OBs and OCs result in a balance between bone formation and bone resorption. OCs and OBs are the core cells involved in bone remodeling. Osteocytes, cytokines, and hormones contribute to this process. OCs are derived from monocytes and reabsorb the bone matrix via enzyme secretion. OBs originate from mesenchymal stem cells and build a bone matrix by secreting collagen. Furthermore, interleukin-6 (IL-6) produced by immature OBs upregulates osteoclasts, while osteoprotegerin (OPG) produced by mature OBs inhibits the activation of OCs. Osteoclastogenesis and osteoblastogenesis contribute to the control of bone remodeling in new bone formation.

Myeloma cells also secrete cytokines to stimulate osteoclastogenesis by interacting with bone marrow stromal cells and the microenvironment. These cytokines include interleukin (IL)-1b, IL-3, IL-6, IL-11, and IL-17, as well as tumor necrosis factor-alpha (TNF-α), C-C motif ligand 3 (CCL3), annexin2, and stromal cell-derived factor-1 alpha (SDF-1α), which also suppress OBs to inhibit bone formation. The uncoupling of osteoclastogenesis and osteoblastogenesis disrupts the bone remodeling process.

**Figure 1:**
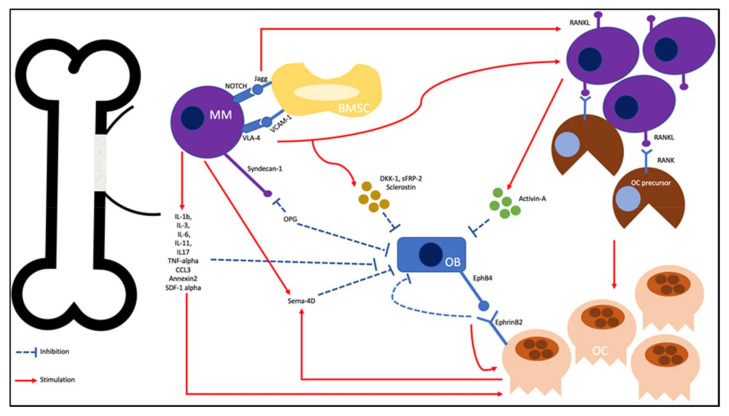
Pathogenesis of multiple myeloma (MM) -related skeletal-related events (SREs).

Osteolytic bone disease is pathognomonic of MM-related SREs. Bone destruction is mediated by increased OC activity and OB inhibition [5]. The microenvironment in myeloma bone disease includes cellular interactions between myeloma cells and bone marrow cells, including bone marrow stromal cells (BMSCs), OBs, and OCs. These interactions promote osteocyte apoptosis, resulting in increased myeloma growth and osteolytic bone destruction (Figure 1) [6,7,8,9,10,11,12,13,14,15,16].

### 2.1. Increasing OC Activity

As a result of the assessments of the mechanisms involved in MBDs, the RANKL/RANK pathway has been identified to play a major role in the development of osteolytic bone disease. RANK and its ligand (RANKL) activate the downstream factor, NF-κB, which simultaneously activates OC differentiation and decreases OC apoptosis. RANKL is mainly expressed by osteoblasts, but it is also expressed by activated lymphocytes, BMSCs, and endothelial cells [6,17,18,19,20,21]. RANKL facilitates OC activation by binding to RANK on the OC membrane. Another influential pathway is the Notch signaling pathway, which promotes OC activity. The interaction of the Notch family in the membranes of MM cells that bind to the Jagged ligands expressed in the membranes of BMSCs results in increased RANKL expression by MM cells [21]. Other factors favoring osteoclastogenesis and OC-mediated bone destruction include chemokines, such as the chemokine C-C motif ligand 3 (CCL3), or macrophage inflammatory protein 1-alpha (MIP1α), SDF-1α, IL-1b, IL-3, IL-6, IL-11, IL-17, annexin 2, and TNF-α [3,21,22,23,24,25,26,27,28,29,30,31,32,33,34,35,36]. The CCL3 chemokine or MIP1α, secreted by myeloma cells, triggers osteoclastogenesis by binding to chemokine receptor type 1 (CCR1) and CCR5 on OOCs. Simultaneously, they facilitate the adhesion between myeloma cells and BMSCs, stimulating increased production of IL-6 and RANKL. Finally, myeloma cells construct a feedback loop to ensure their own growth by producing CCL3 (MIP-1α), and increase OC activity in combination with RANKL and MIP-1α in synergy with IL-6 to promote their survival.

### 2.2. Enhancing OB Inhibition

MBD is complicated by OB inhibition, resulting in bone loss without repair. The Wingless-type (Wnt) pathway is a central regulator of OB differentiation and normal bone homeostasis [24,37]. OCs play a key role in the modulation of remodeling by negatively regulating Wnt signaling through the expression of Wnt inhibitors. Dickkopf-1/2 (DKK-1/2) prevents further bone formation [24,25,37,38]. OCs and myeloma cells produce Wnt signaling inhibitors such as sclerostin (Scl), DKK-1, and the secreted, frizzled-related protein 2 (SFRP-2) to suppress OB activity, resulting in decreased osteoblastogenesis, which contributes to MM-related bone resorption and disease progression. DKK-1 inhibits immature OBs and enables the maximum amount of IL-6 to be secreted, and it also suppresses OB differentiation and activity [25,39]. SFRP-2 is a Wnt antagonist and a soluble factor produced by myeloma cells. Scl inhibits OB development, weakens bone mineralization [33], and impedes Scl production with monoclonal antibodies in preclinical MM models that restore deregulated bone metabolism and decrease bone fragility [15,33,40]. 

The transcription factor, runt-related transcription factor 2 (Runx2)/core-binding factor runt domain alpha subunit 1 (CBFA1), is an important driver of OB differentiation and bone formation. Myeloma cells inhibit osteoprogenitor cells to downregulate the differentiation of OBs by inhibiting Runx2/CBF1A, which results in osteolytic lesions. Furthermore, Runx2/CBFA1 mediates the secretion of OPG. Inhibition of Runx2/CBFA1 decreases OPG and increases osteoclastogenesis. OPG is produced by OBs, BMSCs, and osteocytes. OPG inhibits the interaction between RANKL and RANK. Myeloma cells degrade OPG through the membrane syndecan-1 system to inhibit OB activity [41,42,43].

### 2.3. Bidirectional Signaling in the Uncoupling of Osteoclastogenesis and Osteoblastogenesis

The development of osteolytic lesions is the result of the uncoupling of osteoclastogenesis and osteoblastogenesis. However, several other factors are involved in their development. The interaction between Ephrin type-B receptor 4 (EphB4) in the membranes of OBs and EphinB2 expressed in the membranes of OCs results in enhanced OB inhibition and increased OC activity [44]. The apoptosis of OBs also enriches the expression of Notch and promotes Scl secretion and RANKL expression, which enhances OB inhibition and increases OC activity.

Transforming growth factor beta (TGF-β) and ILs, such as IL-3 and IL-7, are also involved in OB suppression. OCs and myeloma cells further inhibit OBs through semaphorin-4D [36,45,46]. TGF-β is produced by the bone matrix during bone resorption and inhibits osteoblast differentiation. IL-3 and IL-7 play a bidirectional role in inhibiting OBs by inducing activin A and suppressing Runx2, and this inhibits osteoblastogenesis. TNF-α also has a bidirectional activity in osteoclastogenesis and can suppress OB differentiation. TNF-α inhibits OB differentiation by decreasing Runx2, a key regulator of osteoblastogenesis. Furthermore, TNF-α can induce the apoptosis of mature OBs. 

Interestingly, proteasome inhibitors promote OB differentiation independently of Wnt signaling. Thus, they have an anabolic effect on myelomatous bone [47]. MBD development has a direct correlation with the stimulation of OCs and inhibition of OBs.

### 2.4. MM and the Bone Microenvironment

Myeloma cells mainly survive and proliferate in the bone marrow niche, which interacts with the surrounding bone marrow microenvironment. The bone marrow microenvironment includes two compartments that interact with myeloma cells. A non-cellular compartment consists of soluble factors, such as cytokines, chemokines, growth factors, and extracellular matrix proteins such as collagen, fibronectin, and laminin. In contrast, the cellular compartment consists of hematopoietic and non-hematopoietic cells, fibroblasts, OCs, OBs, and BMSCs [42]. 

There is constant crosstalk among different cell subtypes in the bone marrow microenvironment. The homing of MM cells is favored by their adhesion to BMSCs through Notch bidirectional signaling, which facilitates the interactions among MM cells, BMSCs, and OCs. This results in significant changes in the bone marrow microenvironment that promote MM proliferation and bone destruction [43,48,49]. The dysregulation of the EphrinB2/EphB4 pathway in MM also weakens the normal interaction between OCs and OBs, leading to increased bone loss [21,36]. Positive feedback cycles in the interactions between MM cells and the bone microenvironment have been assumed to lead to increased bone resorption and MM cell proliferation through the IL-6 and BMSC adhesion-related pathways [50]. Myeloma cells induce high levels of TNF-α in the marrow microenvironment. TNF-α increases the BMSC production of OC-activating factors, such as RANKL and IL-6, by increasing the transcription factor spliced X-box binding protein 1, thereby increasing osteoclastogenesis.

OCs may participate in the immunosuppressive microenvironment by promoting the expansion of T helper (Th) 17 lymphocytes and myeloid-derived suppressor cells; however, they inhibit the activity of cytotoxic T and natural killer cells against myeloma cells. Interestingly, the interplay between MM cells and mature OBs may provide a unique niche for MM cells to be maintained in a quiescent state, whereas OB dysfunction or OC remodeling of the endosteal niche allows for their reactivation.

## 3. Predictors or Biomarkers

MBD is evaluated using plain radiographs, magnetic resonance imaging (MRI), positron emission tomography (PET) scans, technetium-99m-sestamibi (Mibi) scanning, and dual-energy X-ray absorptiometry (DEXA) scanning, all of which provide comprehensive information. Biochemical markers of bone resorption are also under investigation, despite the limited availability of the above assays. Further, due to the lack of extensive testing in patients, the routine use of these assays is difficult to analyze [51,52,53]. 

An investigation of the molecular basis of MBD to develop predictive markers or to identify patients at high risk of developing SREs during therapy with bisphosphonates (BPs) was carried out with 261 myeloma samples, which were analyzed by global gene expression profiling. Genetic analysis, including the Wnt signaling antagonist DKK1 genes involved in growth factor signaling and apoptosis, and the overexpression of the interferon (IFN)-induced family or factors involved in cell signaling and mitosis, revealed molecules that are significantly associated with SREs. Higher serum calcium levels and the presence of bone disease and hyperdiploidy at presentation were also identified to be associated with a high risk of SRE development. The simple expression-based SRE predictor can effectively identify individuals at high risk of developing bone disease during treatment with BPs. Such predictors could assist in the development of future trials of novel therapies that aim to treat or manage MBD [54].

## 4. Treatment Overview for MBD

When MM and MBD are diagnosed, several treatments are available. However, a multidisciplinary approach is needed to guarantee that a patient’s quality of life is retained, by using analgesics for pain, surgery, or radiotherapy for MBD. MBD is fatal in the absence of adequate anti-MM treatment. Thus, MM-management plans need to consider treating the underlying MM as well as MBD. Preventive therapies are also needed to delay disease progression in MBD. The mainstay treatment involves the use of antiresorptive agents. However, MBD is often treated with radiation therapy, vertebroplasty, surgery, BPs, and anti-RANKL moAb (Table 1).

Recently developed novel anabolic agents that target sclerostin and DKK1, which promote osteoblastogenesis and bone formation and have the potential to repair existing lesions, may lead to a substantial improvement in MBD. The rest of this review is focused on current treatments for MBD and further developments in the treatment of MBD based on its pathogenesis.

### 4.1. BPs

BPs are pyrophosphate analogs that bind to exposed bone areas of hydroxyapatite crystals. BPs inhibit OC activity and function, providing effective therapy for the SREs of MM [61]. BPs are well-established and are the current standard of care for MBD [38,61,62]. There are two types of BPs: non-nitrogen-containing BPs, such as clodronate, which induce OC apoptosis by causing the accumulation of non-hydrolyzable ATP analogs; and nitrogen-containing BPs, such as pamidronate and ZA, that bind to hydroxyapatite and cause OC apoptosis by inhibiting the farnesyl diphosphate synthase enzyme of the mevalonate pathway.

Zoledronic acid (ZA) was demonstrated to be superior in decreasing SREs relative to clodronate in the MRC Myeloma IX trial. Pamidronate and ZA have greater potency in inhibiting the transformation of monocytes to OCs and might facilitate the apoptosis of OCs [55,56,57,58,63]. In large-scale, randomized clinical studies, improved progression-free survival and overall survival by novel BPs in the treatment of MBD has been shown in subanalyses of the overall population. BPs may thus be well-tolerated by patients with MM. The adverse events associated with BPs are mild and easily managed. However, renal function must be continuously monitored. Favorable results of long-term treatment with BPs (Bonefos, Ibandronate) in combination with antitumor therapy were observed in 364 patients. During a 15-year observation period, a median survival of 94 months with a 35% probability of 10-year survival was achieved with a significant decrease in bone complications in 58% of patients in the treatment groups compared to 14% in the placebo group [64].

A recent large-scale investigation examined ZA effectiveness in 111,679 patients with bone metastases from breast cancer or prostate cancer, or MM patients using real-world databases. The findings revealed a decreased risk of SREs in patients with a history of SREs. However, no preventive effects of ZA were observed in patients without this history [65].

Although BPs are the initial, first-line treatment for MBD, their long-term adverse effects limit their use. These adverse effects include renal toxicity (which requires dose reduction in patients with renal impairment), flu-like symptoms, gastrointestinal upset during administration, atrial fibrillation, atypical femoral fracture, and osteonecrosis of the jaw (which can occur in 3.5% of patients). Although the efficacy of clodronate was reportedly inferior to that of ZA, a lower rate of osteonecrosis of the jaw was evident relative to ZA treatment (1 vs. 4%, respectively). Furthermore, pamidronate can be administered to patients with significant renal impairment [57]. Based on the updated results of the Myeloma IX trial, ZA should be administered until patients’ disease progression fails to achieve beneficial partial response. BPs are recommended to be administered for up to 2 years if they are well-tolerated at the time of relapse.

### 4.2. Denosumab

The denosumab anti-RANKL moAb strongly binds to RANKL. The resulting inhibition resembles the effect of endogenous OPG and decreases bone resorption. In 2018, both the United States Food and Drug Administration (USFDA) and European Medicines Agency (EMA) granted the supplemental approval of denosumab for the prevention of SREs in MM patients based on the phase 3 results of the 20090482, randomized, double-blind trial comparing the safety and efficacy of monthly denosumab to monthly ZA in patients with MM. The trial was undertaken to demonstrate non-inferiority and possibly superiority regarding progression-free survival [20,60,66].

No apparent difference between denosumab and ZA has been found regarding overall survival or skeletal events. Further, their safety profiles are very similar. ZA may result in slightly more renal toxicity. However, this is balanced by the higher rates of hypocalcemia with denosumab [66,67]. In MM, there are no data on denosumab cessation, and the drug has been licensed for continuous use. Based on the results of the 10-year follow-up of the Future Revascularization Evaluation in Patients with Diabetes Mellitus: Optimal Management of Multivessel Disease (FREEDOM) and open-label extension studies, the continuous administration of denosumab has a manageable toxicity profile and shows continuous improvement in BMD, with decreased fracture risk among postmenopausal women with osteoporosis, especially among patients at high risk for SREs. 

Denosumab is recommended when BPs cannot be prescribed, for example, due to renal toxicity. There is also a recommendation to use denosumab if hypercalcemia of malignancy occurs and is refractory to BPs. Denosumab is not nephrotoxic and can be administered as a subcutaneous injection, which allows easier access for patients to this treatment and provides a potential alternative to those that cannot tolerate BPs.

### 4.3. ASCT

RANKL and OPG may be crucial in the pathogenesis of bone destruction. BPs may clinically improve skeletal prognosis and survival in patients with myeloma. High-dose chemotherapy with autografting may normalize abnormal bone resorption; however, the effect may take several weeks to emerge and may be paralleled by increased OB activity [68]. ASCT could normalize abnormal bone remodeling by decreasing the ratio of soluble RANKL (sRANKL) and OPG in patients with MM [69].

### 4.4. Bortezomib-Based Regimens

Bortezomib is a proteasome inhibitor used to treat MM and is generally used in combination with other medications [2,70]. Bortezomib-based regimens are indicated for MM in patients who have and have not previously received treatment.

At baseline in one study [71], patients with relapsed MM displayed increased serum concentrations of DKK-1, sRANKL, sRANKL/OPG ratio, C-telopeptide of type I collagen (CTX), and tartrate-resistant acid phosphatase isoform-5b (TRACP-5b), and reduced bone alkaline phosphatase and osteocalcin. Serum DKK-1 levels were correlated with CTX and severe bone disease. Bortezomib administration decreased serum DKK-1, sRANKL, CTX, and TRACP-5b levels after four cycles of therapy, and increased bone alkaline phosphatase and osteocalcin, irrespective of treatment response. Accordingly, bone remodeling was observed to be normalized in relapsed myeloma [71].

## 5. Novel Agents

The high prevalence of osteolytic bone disease in MM highlights the need for novel therapies targeting the bone microenvironment [16,72,73,74,75,76]. Several novel agents are under investigation for their positive effects on bone remodeling mediated by OC inhibition. The downregulation of OB differentiation has prompted the use of anabolic agents. In addition to restoring bone remodeling, these novel agents may impede tumor growth in vivo.

MBD is dependent on the uncoupling of bone remodeling that is provoked by increased bone resorption mediated by OCs. Typically, bone formation is reduced because of the downregulation of the number of functional OBs [77]. Functionally, MM cells interfere with physiological bone remodeling by releasing OC-promoting cytokines, such as RANKL, IL-1, IL-6, CCL3, and CCL20. Moreover, MM cells are also responsible for the inhibition of osteogenesis, as they upregulate OB inhibitors, including DKK1 and Scl [21,35,36].

Therapeutic strategies targeting pathophysiologic interactions between MM cells, OCs, and OBs in the bone marrow microenvironment are key to deferring the onset of SREs, avoiding bone lesions, and achieving tumor regression. BPs and denosumab are bone-modifying agents with anticatabolic properties that are recommended for the treatment of MBD. Other potential therapeutic targets include DKK1 and Scl antagonists [25,28,33]. Standard anti-MM agents, such as proteasome inhibitors, are also known to influence osteolytic lesions [78]. The novel agents under investigation are summarized in Table 2.

## 6. Conclusions

MBD is one of the main causes of death in patients with MM, even in patients in remission. This bone disease is caused by an imbalance in bone remodeling, with increased OC and decreased OB activity and formation, culminating in lytic bone destruction. The survival outcomes and quality of life of MM patients are improved with the administration of new agents. BP and RANKL inhibitors are the current standard of care. However, their limited efficacy, inability to promote new bone formation, and concerns over their safety profile demonstrate the strong potential utility of bone anabolic agents. Although patients’ survival increases with treatment with these inhibitors, it is necessary to introduce more effective agents for the treatment of MBD. As the molecular mechanisms guiding MBD are increasingly well-understood, new therapeutic targets are being broadly investigated in the preclinical setting, and clinical trials with novel agents are yielding promising results. Mounting evidence of the benefits of bone anabolic agents, such as anti-DKK-1, anti-RANKL, antisclerostin, and anti-TGF-β, will herald improvements in the treatment of MBD. With many agents in clinical trials and many target factors identified, combination treatment demonstrates the greatest potential for the management of MBD. The reduction in bone resorption combined with new bone formation is necessary to reduce the burden of disease. Combining antiresorptive agents and antimyeloma therapies may also serve as a future treatment strategy for MBD. Further research is, however, necessary to validate these outcomes in patients and ultimately determine patients’ quality of life and survival.

## Figures and Tables

**Table 1:** Treatment of MM bone disease (MBD).

Study	Study Design	Patient Papulation	Treatment Drug	Treatment Schedule	Patients, n	Median Time to First SRE, months	SREs Incidence, %	ONJ Incidence, %	Renal Toxicity, %
Berenson et al. [55]	Randomization	Stage III myeloma	Pamidronate vs. placebo	90 mg pamidronate 4 h IV infusion every 4 weeks for 9 cycles	196 vs. 181	Significantly less in placebo group (*p* = 0.01)	24 vs. 41 (*p* < 0.01)	NR	NR
Rosen et al. [56]	Phase III, double-blind, comparative trial	Durie-Salmon Stage III myeloma	ZA vs. pamidronate	4 or 8 mg ZA IV or 15 min or 90 mg pamidronate IV 2 h every 3–4 w for 12 months	129 vs. 65	12.5 vs. 9.4	NR	NR	NR
Gimsing et al. [57]	Double-blind, randomized, phase 3 trial	MM patients starting antimyeloma treatment	Pamidronate	30 vs. 90 mg of pamidronate	252 vs. 252	10.2 vs. 9.2 (*p* = 0.63)	33.7 vs. 35.2	0.8 vs. 3.2	NR
Morgan et al. [58]	Computer-generated randomization	Newly diagnosed MM	ZA vs. clodronate	4 mg of ZA IV every 3–4 weeks or 1600 mg of clodronic acid orally daily	981 vs. 979	NR	27 vs. 35 (*p* = 0.0004)	4 vs. 1	Similar for the two treatment groups (*p* = 0.55)
Himelstein et al. [59]	Randomized, open-label	MM with at least one site of bone involvement	ZA	ZA every 12 vs. every 4 weeks	139 vs. 139	NR	55 vs. 60	NR	NR
Raje et al. [60]	Double-blind, double-dummy, randomized, controlled, phase 3	MM with at least one lytic bone lesion	Denosumab vs. ZA	120 mg of denosumab SC plus placebo IV or ZA 4 mg IV plus placebo SC every 4 weeks	859 vs. 859	22.8 vs. 24 (*p* = 0.01)	43.8 vs. 44.6	4.1 vs. 2.8	10 vs. 17.1

BPs, bisphosphonates; MM, multiple myeloma; NR, no report; RANKL, receptor activator of nuclear factor-kappa B ligand; SREs, skeletal-related events; ZA, zoledronic acid; IV, intravenous; SC, subcutaneous.

**Table 2:** Novel agents under investigation.

Molecular Target	Mechanism	Use in MM/Therapeutic Implication
Increased OC Activity		
Inhibition of miR-21 [41]	Expression of miR-21 reduces OPG expression and secretion. MicroRNA (miR-21) overexpression induced by MM–BMSCs interaction antagonizes the physiologic RANKL/OPG balance. OCL activity is dependent on BMSC miRNA-network perturbation. Antagonizing miR-21 may reduce STAT3 signaling mediated by PIAS3 upregulation.	The combination of miR-21 antagonism with conventional drugs might improve the clinical outcome of MM patients.
CCL-3 (MIP-1α) [7,79,80]	CCL3 inhibits OB function and contributes to OB/OC imbalance by inhibiting OB differentiation and function in MBD. OCs secrete high levels of CCL3, which triggers MM cell migration.	CCL3 antibody partially restores OB activity through the upregulation of the OCN, Runx2, and Osx. MLN3897, a novel CCR1 inhibitor, impairs osteoclastogenesis and inhibits the interaction of MM cells and OCs by inhibiting Akt signaling and abrogates MM cell-to-OC adhesion to inhibit MM cell survival and proliferation.
Activin A [81,82]	Activin A is produced in MM-related osteolysis. Lenalidomide acts directly on BMSCs via an Akt-mediated increase in the c-Jun N-terminal kinase-dependent signaling, resulting in activin A secretion, with the consequent inhibition of osteoblastogenesis.	Lenalidomide + Activin A inhibitor. Phase 1 clinical trial. Sotatercept (ACE-011) (ligand trap fusion receptor) is a recombinant activin receptor type IIA IgC-Fc fusion protein to prevent continued loss of bone. It causes an increase in hemoglobin, hematocrit, and red blood cell counts in patients with myeloma. Phase 2 clinical trial completed showing that sotatercept increased BMD in MM patients.
IL-6 [83]	In the bone marrow microenvironment, IL-6 is produced by BMSCs, mediating MM cell growth and preventing apoptotic cell death. IL-6 triggers at least three major signaling pathways: Ras/MEK/ERK cascade, JAK2/signal transducer and activator of transcription (STAT-3) cascade, and PI3K/Akt cascade. IL-6 protects against apoptotic cell death induced by a variety of agents, including dexamethasone. IL-6 controls the expression of various other key growth and survival mechanisms in myeloma.	Anti-IL-6 moAb exhibits anti-MM activity in clinical trials.
IL-17 [84,85]	IL-17 is significantly elevated in blood and bone marrow in MM, and IL-17A promotes MM cell growth via the expression of IL-17 receptor and induces IL-6 production.	AIN457, anti-human IL-17A human moAb in MM significantly inhibited MM cell growth OC cell differentiation.
**Suppressed OB Activity**		
Wnt pathway [37,86]	Wnt3a signaling within bone inhibits MBD and tumor growth.	Treatment of myelomamatous SCID-hu mice with recombinant Wnt3a-stimulated bone formation and attenuated MM growth. LGR4 expression allows MMs to respond to (pre)OB-derived R-spondins (RSPOs), resulting in stabilization of the Wnt receptors and markedly enhances sensitivity to auto and paracrine Wnt ligands. These results provide further support regarding the potential anabolic effect of the targeting of proximal Wnt signaling in MM.
Scl [15,33,87,88]	Scl, an osteocyte-derived inhibitor of Wnt/β-catenin signaling, is elevated in MM patient sera and increased in osteocytes in MM-bearing mice.	Administration of anti-Scl antibody (Scl-Ab) increased bone mass and decreased osteolysis in immune-competent mice with established MM. Sost/Scl inhibition increased OB numbers, stimulated new bone formation, and decreased OC number in MM-colonized bone. Romosozumab is an anti-Scl moAb for benign bone disorders.
DKK1 [31,89,90]	DKK1 is another antagonist of the Wnt signaling pathway secreted by MM cells. By binding to LRP6, it inhibits osteoblastogenesis and new bone formation. DKK1 is also responsible for enhanced Scl secretion in the bone microenvironment, as Scl is released by immature OBs in the presence of MM-derived DKK1.	BHQ880, a DKK1 neutralizing Ab, increased bone anabolic activity in a phase 2 clinical trial.
EphrinB2/EphB4 signaling pathway [44]	Bidirectional signaling between the cell surface ligand ephrinB2 and its receptor, EphB4, is involved in the coupling of osteoblastogenesis and osteoclastogenesis and in angiogenesis.	The ephrinB2/EphB4 axis is dysregulated in osteoprogenitors from myeloma patients. Its activation affects myeloma bone disease and tumor growth.
Adiponectin [91]	Patients who subsequently progressed to myeloma have a lower serum adiponectin concentration. The apolipoprotein peptide, mimetic L-4F, was used for the pharmacologic enhancement of adiponectin. L-4F reduced tumor burden, increased the survival of myeloma-bearing mice and prevented myeloma bone disease.	A novel mechanism results in a decrease in host-derived adiponectin and promotes myeloma tumor growth and osteolysis. Increasing adiponectin may have potential therapeutic benefits for the treatment of myeloma and the associated bone disease.

BM, bone marrow; BMSC, bone marrow stromal cell; CCL3, chemokine C-C motif ligand 3; DKK1, Dickkopf-1; IL-6, Interleukin-6; IL-17, Interleukins 17; IL-17A, Interleukins 17A; mAb, monoclonal antibody; MBD, myeloma bone disease; MM, multiple myeloma; OB, osteoblast; OC, osteoclast; OPG, osteoprotegerin; RANKL, receptor activator of nuclear factor-kappa B ligand; Scl, sclerostin; Wnt, Wingless-type6.

## Abbreviations

ASCT	autologous stem cell transplantation
BMA	bone-modifying agents
BMSC	bone marrow stromal cell
BP	bisphosphonate
CCL3	chemokine C-C motif ligand 3
CTX	C-telopeptide of type I collagen
DEXA	dual-energy x-ray absorptiometry
DKK-1	dickkopf-1
IL	interleukin
MBD	MM bone disease
Mibi	technetium-99m-sestamibi
MM	multiple myeloma
MRI	magnetic resonance imaging
OB	osteoblast
OC	osteoclast
OPG	osteoprotegerin
PC	prostate cancer
PET	positron emission tomography
RANKL	receptor activator of nuclear factor-kappa B ligand
Scl	sclerostin
sRANKL	soluble receptor activator of nuclear factor-kappa B ligand
SREs	skeletal-related events
TRACP-5b	tartrate-resistant acid phosphatase isoform-5b
Wnt	Wingless-type
ZA	zoledronic acid
